# When Trustworthiness Meets Face: Facial Design for Social Robots

**DOI:** 10.3390/s24134215

**Published:** 2024-06-28

**Authors:** Yao Song, Yan Luximon

**Affiliations:** 1 College of Literature and Journalism, Sichuan University, Chengdu 610065, China; yao.song@scu.edu.cn; 2 Digital Convergence Laboratory of Chinese Cultural Inheritance and Global Communication, Sichuan University, Chengdu 610065, China; 3 School of Design, The Hong Kong Polytechnic University, Hong Kong 999077, China

**Keywords:** eye shape, mouth shape, trustworthiness perception, robot attitude, social robot

## Abstract

As a technical application in artificial intelligence, a social robot is one of the branches of robotic studies that emphasizes socially communicating and interacting with human beings. Although both robot and behavior research have realized the significance of social robot design for its market success and related emotional benefit to users, the specific design of the eye and mouth shape of a social robot in eliciting trustworthiness has received only limited attention. In order to address this research gap, our study conducted a 2 (eye shape) × 3 (mouth shape) full factorial between-subject experiment. A total of 211 participants were recruited and randomly assigned to the six scenarios in the study. After exposure to the stimuli, perceived trustworthiness and robot attitude were measured accordingly. The results showed that round eyes (vs. narrow eyes) and an upturned-shape mouth or neutral mouth (vs. downturned-shape mouth) for social robots could significantly improve people’s trustworthiness and attitude towards social robots. The effect of eye and mouth shape on robot attitude are all mediated by the perceived trustworthiness. Trustworthy human facial features could be applied to the robot’s face, eliciting a similar trustworthiness perception and attitude. In addition to empirical contributions to HRI, this finding could shed light on the design practice for a trustworthy-looking social robot.

## 1. Introduction

As a technical application in artificial intelligence, a social robot is one of the branches of robotic studies that emphasizes socially communicating and interacting with human beings [1,2,3]. Distinct from other humanoid and industrial robots, which usually have limited human-like features, the state-of-art (SOTA) social robots are usually equipped with a screen-based head interfaced with an animated or human-like face to respond to and meet the needs of human beings [4,5]. In fact, a human-like head for a social robot plays a significant role in facilitating interaction in the human–robot relationship [6,7] since human beings might adapt their previous related experience and recognition to interact with such emerging creations [8].

Similar to interpersonal interaction, trustworthiness towards a social robot is crucial in human–robot interaction since the social robot, in daily life, usually acts as a “listener and responder”, providing not only practical assistance but also emotional support for human beings [9]. In this view, it is important that it should be considered a trustworthy “partner or friend” [10]. However, to communicate trustworthiness in a social robot is a challenging problem: there are at least three potential factors that could influence trustworthiness evaluation for social robots [11]. For example, robot-related factors (i.e., the related features or designs of the social robot), human-related factors (the particular need or expectation of human beings), and applied scenario-related factors (the specific environment in the given task implementation) all could have an impact on human–robot interaction [12]. Within these influential factors, robot-related factors, such as robotic features, are considered the most significant factor in improving trustworthiness towards a social robot [13,14].

Humans have the instinct and ability to recognize a face or face-like goods, such as a car, clock, or plug [15]. Todorov further implied people could finish multi-level personality evaluations, such as trustworthiness, competence, and aggressiveness, in a short time [16]. The evolutionary adaptation in the history of human beings might account for people’s conscious or unconscious intention to search for faces, no matter in humans or objects: the face is a strong attention-catching stimulus that might be processed simultaneously [17]. When seeing a robot’s face, people might try to make the analogy between human characteristics and robotic features, helping them to perceive its human-like features [18,19,20]. In addition, the literature has also suggested the impact of the adaptation of a product or virtual character face with human characteristics on people’s attitudes and other social perceptions. For instance, human-like virtual agents are more likable, appropriate, and trustworthy [21], and a social robot with head-like interfaces might be evaluated more positively [19].

Within the investigation of facial trustworthiness, eye and mouth shape are crucial factors in deciding trustworthiness [22,23]. However, limited prior research has shed light on the specific eye and mouth of a social robot’s face. Although both robot and behavior research have realized the significance of social robot design for its market success and the related emotional benefit to users [4,15], the specific design of the eye and mouth shape of a social robot in eliciting trustworthiness has still received only limited attention. In order to address this research gap, the current study tries to validate whether trustworthy perception towards a social robot shares the same features in the eye and mouth with humans, and we further discuss the mediating role of trustworthiness in attitude evaluation.

## 2. Literature Review

Humans have a long tradition of interacting with robots and have shown great acceptability towards robots with head-like features [19,20]. The reason for this preference might lie in human beings themselves; it could be their biological feature, cephalization, in which physical organs and neural systems tend to concentrate in the upper part of the body [24,25]. From the perspective of evolution, the organization of these biological features could provide animals and humans not only physical advantages (i.e., maintaining temperature) but also social benefits (i.e., easy to express signals). Although robots are artificial creatures made by humans, they could still enjoy evolutionary advantages by being designed with head-like features [19].

When encountering one person or seeing a robot, we might form a mental image of them quickly. This is the so-called first impression, and it is important in our daily lives; it not only occurs in human perception but also takes place in forming an attitude towards a person or social robot [18,20,26]. Additionally, it happens unconsciously, is hard to recognize, and influences our decision-making process [27,28]. Prior research has discussed the positive attitude and social perception towards specific appearance in the context of humans, suggesting a rule-of-thumb: “beauty premium” and “plainness penalty” [29].

To be more specific, facial features are strong predictors for the first impression, which work as significant indicators to form a mental image and make an initial evaluation of one’s attributes [30]. People with specific facial features tend to have a higher possibility of being trusted, of being liked, and eventually of getting more actual benefits [29,31]. This “halo” effect of particular facial features could be interpreted by people’s subconscious facial feature processing: specific features could contribute to various positive interpretations or expectations about others [32]. For example, some facial features, such as a warm smile, could be considered social signals indicating friendliness, capability, confidence, and distinction [31].

Among various social perceptions, trustworthiness towards humans or products plays a crucial role in human relationships, product evaluation, and related behavioral responses [6,33,34]. Indeed, humans are skilled in evaluating facial trustworthiness based on physical traits [35]. For example, Todorov and his colleagues [36] suggested people could finish the initial evaluation of facial trustworthiness within 100 ms, which is more efficient than other social attribution evaluations, such as dominance and attractiveness. In addition, Wout and Sanfey [37] suggested people have a higher intention to interact, cooperate, and invest with trustworthy-looking individuals in an interactive, risky decision-making game.

Previous research has long focused on facial trustworthiness features. Indeed, people tend to share a common evaluation strategy for facial trustworthiness since facial trustworthy features seem to converge across different races and regions [38,39,40]. For example, specific features of the eyes [41], mouth [42], nose [43], the brow–nose–chin ratio [43], hair [42], and even ears [42] have been proven to be strong indicators for predicting facial trustworthiness. Among those features, the eye, nose, and mouth are the most attention-attracting facial properties in evaluating trustworthiness [33,42,44]. For example, Ramanathan et al. [45] introduced an eye-tracking-based facial perception study to illustrate that eye fixations are not focused at the center of facial geometry but spread across facial organs such as the eyes, nose, and mouth.

To be more specific, the eye region is considered one of the most significant features that could influence people’s evaluation of trustworthiness, both for humans and products [15,41,42,46,47,48,49,50]. This region has several specific attributes that could communicate trustworthiness, such as eye size, eye shape, eye gaze, eye color, and eyebrow [42]. Studies on eye shape suggest that people with round eyes (vs. narrow) [51,52] are perceived to be more trustworthy since these characteristics all shared and enjoyed the baby-face appearance traits from an evolutionary perspective [53,54]. Similarly, the mouth region is also perceived to have significant features that have an impact on people’s evaluation of trustworthiness. Prior research has speculated that the central facial properties (mouth and nose region) [55] were significantly positively correlated with attention and trustworthiness. As for the shape of the mouth, there are generally three types of mouths in the literature: an upturned mouth (smiling mouth), a downturned mouth (sad mouth), and a neutral mouth [15]. Regarding this, there is a significant difference in the perceived social attributes among these three scenarios: human face or product “facial” appearance with an upturned or neutral mouth (vs. downturned) was believed to be more trustworthy, friendlier, and attractive [15,18,41,56].

Regarding the consequence of trustworthiness, numerous research studies have proven the effect of trustworthiness on attitude towards the person or object. For instance, the effect of source trustworthiness tended to have a significant impact on the persuasiveness and attitude towards the message: the opinions would receive more attention and be more persuasive if the communicator was believed to be more trustworthy, resulting in a more positive attitude [57]. Seymour and Dolan [58] also suggested that trustworthy endorsers would more effectively deliver persuasive information, contributing to positive attitude formation. Thus, it might be possible that a trustworthy-looking robot might have a higher probability of enjoying a general positive attitude, a robot attitude, which refers to a specific observed subject’s evaluation and state of likes and dislikes towards a robot [59].

Another factor worth noting is the age of respondents. Compared with young people, previous studies have suggested old people might be less sensitive to facial trustworthiness, resulting in a higher trustworthiness evaluation, especially in the first impression [35,60]. Thus, age might act as a covariate with eye and mouth shape, jointly influencing people’s trustworthiness evaluation.

**Figure 1:**
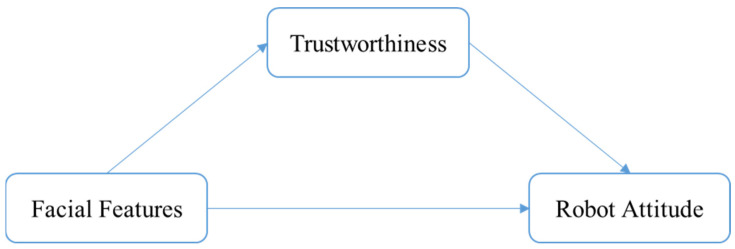
The theoretical model of the current study.

Although prior research has discussed the effect of facial biological features, such as eye shape, mouth shape, positioning, and movement, on trustworthiness evaluation, the majority of research has concentrated on the context of human facial perception. Limited research has addressed the relationship between specific facial features of the robot and its trustworthiness evaluation. Similar to human facial features, the facial features of a social robot also refers to the size, shape, positioning, and movement of a facial organism [61]. McGinn [19] suggested a social robot would be ideally equipped with head-like features that could provide social interaction through facial expressions, gaze interaction, and attention attraction and, eventually, be perceived to be like a real human or a partner in human–robot interaction. However, to date, it is unclear whether there is a “halo” effect in social robots’ perception and whether the rules in human facial perceptions could be applied and still work as significant drivers for trustworthiness evaluation for social robots. Thus, it is both theoretically and practically important to explore the effect of specific facial features, such as eye and mouth shape, on a robot’s trustworthiness evaluation and, more generally, robot attitude in human–robot interaction. Based on the literature above, we might have a theoretical model (see Figure 1) and the following hypotheses:

**H1a.**  *People tend to have a higher trustworthiness perception towards a robot with round eye design (vs. narrow eye design)*.

**H1b.**  *People tend to have a higher level of attitude towards a robot with round eye design (vs. narrow eye design)*.

**H1c.**  *Perceived trustworthiness mediates the effect of eye shape on robot attitude*.

**H2a.**  *People tend to have a higher trustworthiness perception towards a robot with an upturned or a neutral mouth design (vs. downturned mouth design)*.

**H2b.**  *People tend to have a higher level of attitude towards a robot with an upturned or neutral mouth design (vs. downturned mouth design)*.

**H2c.**  *Perceived trustworthiness mediates the effect of mouth shape on robot attitude*.

## 3. Methodology

**Figure 2:**
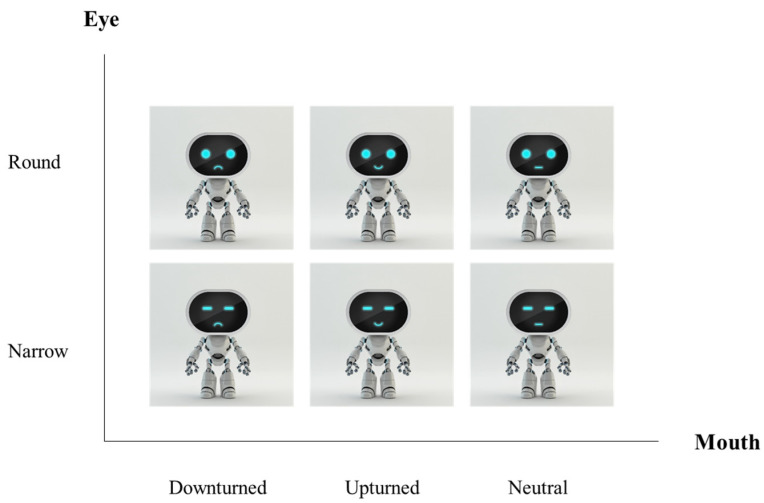
Mouth and eye shape interaction on trustworthiness evaluation.

We conducted a 2 (eye shape) × 3 (mouth shape) between-subject experiment design that included six scenarios: two eye shapes (round vs. narrow) and three mouth shapes (upturned vs. neutral vs. downturned). A robot designer made all the robot stimuli (Figure 2). In cooperation with a designer, we carefully controlled the potential confounders to avoid influences from an existing social robot or other related fields. Additionally, we kept other robotic features, such as body height, width, positioning, posture, color, and background, unchanged.

In order to analyze the relationship stated above, we recruited a sample from Amazon Mechanical Turk (AMT) in the current study. AMT is a web-based platform that helps to recruit registered workers to finish given work for compensation [62]. A large amount of research in various disciplines has been conducted via this platform because it is reliable [63], accurate [64], effective [65], and diverse [62]. Thus, we consider it might be appropriate and reliable to recruit subjects using this platform to get a better understanding of trustworthiness and attitudes towards the social robot.

A total of 211 participants took part in this experimental study (mean age = 35.74, SD = 10.7; 112 male and 99 female). After consenting to participate, people were randomly selected to be exposed to one of six stimuli and were asked to fill in the questionnaire: 34 participants were exposed to a robot with round eyes and a neutral mouth (mean age = 35.41, SD = 10.30); 36 participants were exposed to a robot with round eyes and an upturned mouth (mean age = 37.01, SD = 11.74); 35 participants were exposed to a robot with round eyes and a downturned mouth (mean age = 35.43, SD = 9.24); 36 participants were exposed to a robot with narrow eyes and a neutral mouth (mean age = 35.42, SD = 12.83); 33 participants were exposed to a robot with narrow eyes and an upturned mouth (mean age = 36.70, SD = 10.21); and 37 participants were exposed to a robot with narrow eyes and a downturned mouth (mean age = 34.62, SD = 9.97). As for the measurement items, trustworthiness was estimated by five items on a nine-point Likert scale (credible; sincere; honest; believable; convincing) [66] and robot attitude was estimated by three items on a nine-point Likert scale (desirable; good; pleasant) [67].

## 4. Analysis and Results

**Figure 3:**
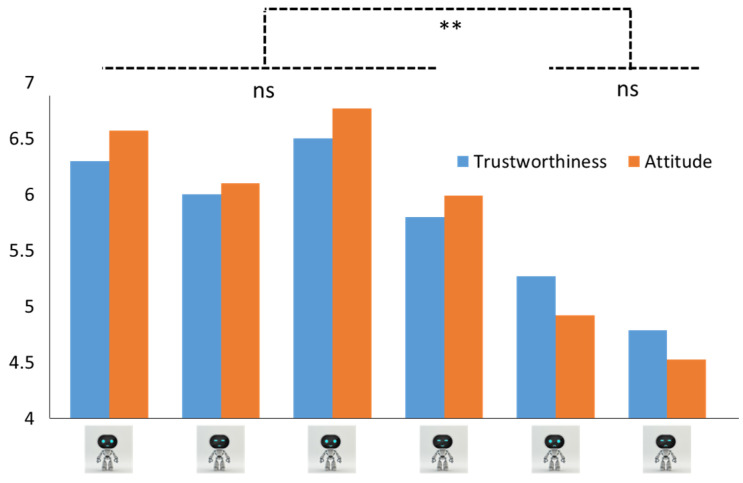
The trustworthiness and attitude evaluation for six scenarios. Note: ** means significant < 0.05; ns means non-significant.

**Figure 4:**
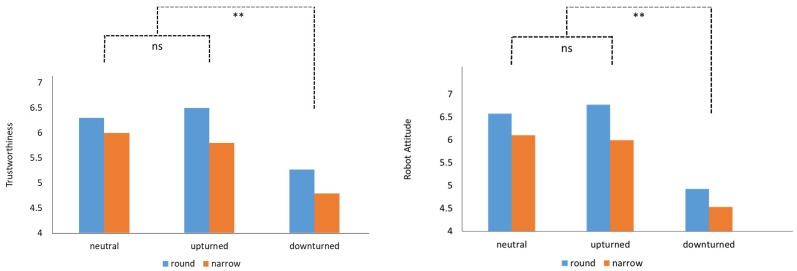
The effect of mouth and eye shape on trustworthiness (**left**) and attitude (**right**) towards the social robot. Note: ** means significant < 0.05; ns means non-significant.

A two-way ANCOVA was conducted with eye shape (round vs. narrow) and mouth shape (upturned vs. neutral vs. downturned shape) as the independent variables, age as the covariate, and perceived trustworthiness as the dependent variable. To specify, the Cronbach’s alpha coefficients showed a high internal consistency of five items (0.939), indicating a high consistency of the current measurement. The results showed that the main effect of mouth and eye design on trustworthiness evaluation was significant, while the interaction effect was not significant (see Figure 3). To be more specific, people in the round eyes scenario (mean = 6.02 vs. 5.51, SD = 1.69 vs. 2.14) showed significantly higher trustworthiness than those who were exposed to the robot with narrow eyes (F(1, 204) = 3.94, *p* < 0.05). In addition, people in the upturned and neutral mouth scenario tended to have a higher trustworthiness evaluation than those in the downturned mouth scenario (mean = 6.16 vs. 6.15 vs. 5.02; SD = 1.80 vs. 1.67 vs. 2.11, respectively; F(2, 204) = 9.20, *p* < 0.05) while there is no statistically significant difference between the upturned and neutral mouth scenarios (*p* = 1.00). Also, the covariate variable, age, was significant (F(1, 204) = 6.22, *p* < 0.05), and there is no interaction effect between mouth and eye on trustworthiness evaluation (F(2, 204) = 0.24, *p* = 0.78). Thus, H1a and H2a were supported (Figure 4 left).

As for the robot attitude, results showed people in the round eyes scenario (Mean = 6.09 vs. 5.52, SD = 1.77 vs. 2.25) similarly showed higher robot attitude than those in the narrow eyes scenario (F(1, 204) = 4.50, *p* < 0.05). Additionally, people in the upturned and neutral mouth scenario tended to have a higher robot attitude than those in the downturned mouth scenario (mean = 6.33 vs. 6.40 vs. 4.72; SD = 1.65 vs. 1.81 vs. 2.12, respectively; F(2, 204) = 18.25, *p* < 0.05) while there was no statistically significant difference between the upturned and neutral mouth scenarios (*p* = 0.97). Moreover, the covariate variable, age, was marginally significant (F(1, 204) = 3.14, *p* = 0.08), and there was no interaction effect between mouth and eye on robot attitude (F(2, 204) = 0.19, *p* = 0.83; see Figure 4, right). Thus, H2a and H2b were supported accordingly.

In order to test H1c and H2c, the mediation role of trustworthiness, we regressed the robot attitude on two facial features separately (eye and mouth) with age as a covariate variable through PROCESS SPSS macro (Model 4, n = 5000 resamples; [68]).

**Figure 5:**
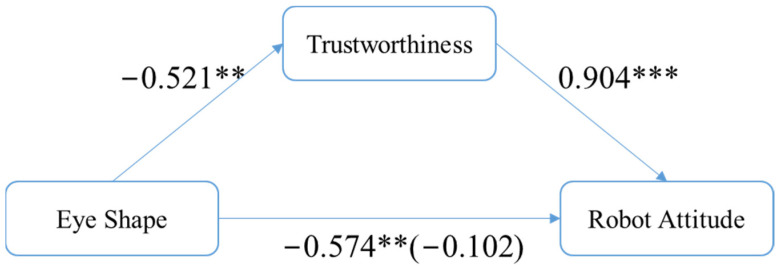
Trustworthiness mediates the effect of eye shape on robot attitude. Note: *** means significant < 0.01; ** means significant < 0.05.

To examine H1c, eye shape was a significant predictor of trustworthiness, with β = −0.521, SE = 0.263, *p* < 0.05 (note: β, hereinafter, stands for the coefficient of regression, while SE stands for standard error of estimate), and it was also a significant predictor of robot attitude (total effect), with β = −0.574, SE = 0.279, *p* < 0.05. In addition, trustworthiness was a significant predictor of robot attitude, with β = 0.904, SE = 0.038, *p* < 0.01. When the mediator, trustworthiness, was controlled, eye shape case was not a significant independent variable of robot attitude (direct effect), with β = −0.102, SE = 0.146. The covariate variable, age, was also not significant (β = 0.006, SE = 0.006, *p* = 0.32). Furthermore, the indirect effect was significant (β = −0.471, SE = 0.245, LLCI = −0.976, ULCI = −0.011 (note: LLCI/ULCI stands for lower/upper level of confidence interval)). Thus, H2c was supported (see Figure 5).

**Figure 6:**
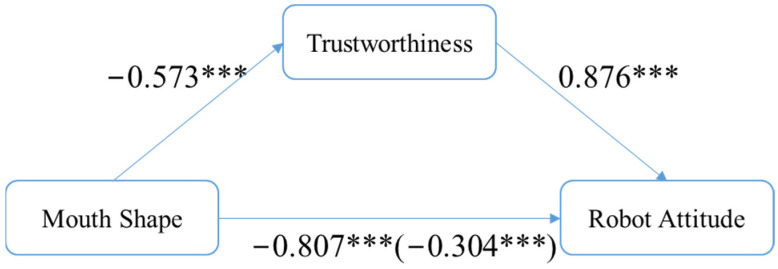
Trustworthiness mediates the effect of mouth shape on robot attitude. Note: *** means significant < 0.01.

To examine H2c, the results showed that mouth shape was a significant predictor of trustworthiness (β = −0.573, SE = 0.156, *p* < 0.01), and it was also a significant predictor of robot attitude (total effect), with β = −0.807, SE = 0.161, *p* < 0.01. Moreover, the trustworthiness was a significant predictor of robot attitude (β = 0.876, SE = 0.037, *p* < 0.01). When the mediator, trustworthiness, was controlled, mouth shape was also a significant predictor of robot attitude (direct effect), with β = −0.304, SE = 0.088, *p* < 0.01. The covariate variable, age, was not significant (β = 0.006, SE = 0.006, *p* = 0.37). Again, we could find a significant indirect effect (β = −0.502, SE = 0.146, LLCI = −0.794, ULCI = −0.208). Accordingly, H2c was supported (see Figure 6).

## 5. Conclusions and Discussion

The social robot, as one of the latest applications in artificial intelligence (AI), might socially meet people’s physical and emotional demands [14,69,70], and people tend to show a more preferable attitude towards social robots with head-like features [19,20]. Indeed, certain facial features might enjoy not only physical advantages but also social benefits because of the evolutionary and genetic basis of human beings [19]. Although trustworthiness is one of the most fundamental social attributions, and numerous research studies have explored the relationship between specific facial features and trustworthiness perception [22,33,43], the majority of prior research has focused on facial trustworthy features in the context of humans; facial trustworthy features for social robots have largely been neglected. Trustworthiness towards social robots also plays a crucial role in human–robot interaction; this research tries to address this research question by examining the effect of specific facial features, eye and mouth shape, on robot trustworthiness evaluation and related robot attitudes. According to the results, this research validated that (1) round eyes (vs. narrow eyes) and an upturned-shape mouth or neutral mouth (vs. downturned-shape mouth) for social robots could significantly improve people’s trustworthiness evaluation in social robots; (2) round eyes (vs. narrow eyes) and an upturned-shape mouth or neutral mouth (vs. downturned-shape mouth) for social robots could also significantly improve people’s attitudes towards robots; (3) the effect of eye and mouth shape on robot attitude was mediated by trustworthiness; (4) there was no interaction effect between eye and mouth shape on trustworthiness evaluation.

There are several contributions to this research. To begin with, prior research on facial trustworthiness was mainly within the context of human facial properties; thus, only a limited number of research studies have tried to figure out whether the rules or conclusions could be applied in designing facial features for social robots. For example, recent work has tried to discuss whether the social robot should have a head-like feature and its impact on social perceptions [19]. Nevertheless, this work mainly focused on the general morphology of social robot design, which neglected numerous traditional explorations in facial trustworthiness. Indeed, trust or trustworthiness, as one category of personality, does not belong to humans exclusively. Instead, it is a symbol by which people convey their expectations to themselves and to others, and this symbolic meaning is known to influence people’s preference for a product or even a robot [71]. In this view, it might be significant to explore whether we could apply previous works on facial trustworthiness to the facial design for a social robot. Through a behavioral experiment approach, the current study has suggested facial trustworthiness features could be applied to social robot design and improve people’s trustworthiness and attitude towards the social robot.

In addition, prior research on facial trustworthiness has suggested people might consider round eyes (vs. narrow eyes) as strong indicators of baby-face appearance traits [53,54], thus improving trustworthiness [51,52]. Similarly, compared with a downturned mouth (sad mouth) and a neutral mouth [15], people with an upturned mouth (smiling mouth) were believed to be more trustworthy and friendlier [15,18,41,56]. However, it was unclear whether the rules might work in the facial design of the social robot and influence the related social perceptions. Consistent with the previous conclusion, the current research, for the first time, provided the initial evidence to prove social robots with round eyes and an upturned mouth (or neutral mouth) could improve people’s trustworthiness and attitude towards the robot. Last, this study tried to contribute to the literature on the evaluation of a social robot by analyzing the underlying mechanism within this phenomenon. Even though we already know the eye and mouth shape might have an impact on perceived trustworthiness and robot attitude, we still need to have a deep understanding of this process and the relationship between different factors. Based on theoretical deduction and empirical results, this study has shown the effect of facial features on robot attitude is mediated by trustworthiness. Regarding the covariate variable, results showed that age was a marginal significant variable in predicting perceived trustworthiness and purchase intentions, suggesting older people tended to have a marginally higher level of perceived trustworthiness and purchase intention in the first impression, which is consistent with prior research [60].

This study also has some practical implications for industrial design practice. Although the current market has various social robots, and some of them even won some honors [72], detailed and specific guidelines are still missing in the current industry, to some extent, such that companies design a social robot primarily relying on their own understanding and intuition [73]. Regarding the risk in intuition-based design that it might dampen user experience and the potential market performance [74], this research provides preliminary instructions for designing a trustworthy social robot so that designers might enjoy intuition from previous findings on human facial trustworthy cues. In this study, we have shown a social robot with a rounded-eye design and an upturned mouth (or a neutral mouth) might enjoy a higher trustworthiness perception, robot attitude, and the probability of commercial success and industrial application. In a future study, we will try to validate other human facial features, such as face shape, and discuss more detailed guidelines to design a trustworthy-looking robot.

There are some limitations that should be addressed in further studies. First, while the current research establishes the foundational effects of facial features on initial trustworthiness impressions prior to interaction, the scenarios used were relatively context-free [75]. People’s perceptions of a robot’s trustworthiness and their attitudes towards it may potentially vary depending on the specific applied interaction scenario and use case [76]. For example, Song and his colleagues have argued that the regulatory fit of contextual cues and dynamic expressions for a social robot could evoke higher levels of trustworthiness [5], though it still examined the general effect of facial features without incorporating detailed interaction scenarios, such as nursing homes [77]. Future work should systematically examine whether these effects persist across different real-world contexts, such as interactions with companion robots [78], healthcare robots [79] for diverse patient populations, educational robots for children versus adults [80], and other applications. Additionally, individual differences among users like age [81], health status [82], technological familiarity [75], and other factors could potentially moderate the observed effects and should be investigated. Previous research has indeed shown age and health factors can influence technology attitudes [83], but that literature centers more on extended interactions rather than initial impressions. The current paper focuses specifically on the first stage—how static facial features like eye and mouth shape influence the all-important initial judgment of trustworthiness before any interaction occurs [7,76]. We examined this baseline effect without considering the potential moderating influences of individual traits [76]. Building on these results, future studies could especially explore how different user characteristics, like age, health status, tech familiarity, etc., may heterogeneously impact trustworthiness perceptions arising from facial appearances.

## Data Availability

The data used in this study are available upon request from the corresponding author.
